# Is Baseline Vertical Implant Positioning Relative to Adjacent Teeth Associated with Peri-Implant Outcomes? A Systematic Review and Meta-Analysis

**DOI:** 10.3390/dj14080523

**Published:** 2026-08-16

**Authors:** Paolo De Angelis, Giuseppe De Rosa, Iacopo Cioccoloni, Alberto Staffieri, Gioele Gioco, Romeo Patini, Luca Raffaelli, Carlo Lajolo

**Affiliations:** 1 Department of Head and Neck and Sense Organs, School of Dentistry, Fondazione Policlinico Universitario Agostino Gemelli IRCCS, Università Cattolica del Sacro Cuore, 00168 Rome, Italycarlo.lajolo@unicatt.it (C.L.); 2 Private Practice, 73100 Lecce, Italy; 3 Private Practice, 76123 Andria, Italy

**Keywords:** dental implants, dental prosthesis, implant-supported, single-tooth, marginal bone loss

## Abstract

**Background**: Vertical implant positioning may influence peri-implant health outcomes and long-term stability; however, the impact of baseline vertical implant positioning relative to adjacent teeth remains unclear. The aim of this systematic review and meta-analysis was to assess the association between baseline vertical implant positioning and peri-implant clinical and radiographic outcomes. **Methods**: The Cochrane Central Register, PubMed, SCOPUS, and Web of Science were searched. Vertical implant positioning relative to adjacent teeth was assessed using two radiographic reference distances: implant platform to cemento-enamel junction (IP–CEJ) and implant platform to adjacent interproximal alveolar bone level (IP–ABL). The primary outcome was peri-implant marginal bone loss (MBL). Secondary outcomes included implant survival and success rates, peri-implant mucositis and peri-implantitis prevalence. Risk of bias was assessed using the Moga checklist, and certainty of evidence was evaluated according to GRADE. **Results**: Four studies including 267 implants fulfilled the inclusion criteria. The pooled mean baseline IP–CEJ and IP–ABL distances were 4.69 mm (95% CI 2.05 to 7.32) and 2.13 mm (95% CI 1.29 to 2.98), respectively. The pooled mean marginal bone loss after at least 12 months, based on three studies, was 0.21 mm (95% CI −0.28 to 0.70). **Conclusions**: The available evidence is insufficient to determine whether baseline IP–CEJ or IP–ABL distances are associated with peri-implant outcomes or to identify an optimal range of vertical implant positioning. The findings should be interpreted cautiously because the certainty of evidence is very low, highlighting the need for prospective studies with long-term follow-up.

## 1. Introduction

Dental implants represent a reliable and widely used option for replacing missing teeth, with reported survival rates for implant-supported fixed dental prostheses (FDPs) of 95.6% at 5 years and 93.1% at 10 years [1,2,3]. It is estimated that the proportion of patients with implant-supported restorations has clearly increased in recent years, rising from 0.7% in 1999–2000 to 5.7% in 2015–2016, with a continued upward trend projected to reach approximately 23% by 2026 [1,2,3,4].

In recent years, the scientific community has increasingly focused on the prognostic factors for implant success that include: patient-related factors (e.g., systemic diseases, pharmacological therapies, and lifestyle habits), implant-related characteristics (e.g., surface, connection type, and design), and anatomical factors of the recipient site (e.g., bone features, bone volume, and keratinized mucosa). It is essential to consider the risk factors that may influence long-term stability: among these variables, the three-dimensional spatial relationship between implants and adjacent teeth has become relevant [5]. Consequently, optimal implant positioning is mandatory to ensure long-term success and patient satisfaction, as it promotes the formation of a stable peri-implant soft tissue seal guaranteeing a cleansable restoration [6,7].

Recently, the vertical distance between the implant platform (IP) and the cementoenamel junction (CEJ) of the adjacent tooth and the vertical distance between the IP and the interproximal bone level of the adjacent tooth (ABL) seem to be clinically relevant variables for long-term success [8]. Different vertical implant positioning may present significant biological and prosthetic challenges: deep mucosal tunnels, incongruous prosthetic designs and emergence profiles, increased prosthetic height, and three-dimensional prosthetic compensation to resolve the anatomic volumetric discrepancy [9,10]. The correct vertical positioning of the implant is crucial to ensure adequate prosthetic and hygienic design through a morphology that facilitates home-care procedures and preserves hard and soft tissue architecture; when the vertical implant position is increased, a deep mucosal tunnel (≥3 mm) may be created. Serino and Ström demonstrated that, in a group of patients affected by peri-implantitis, 48% of the cases were associated with limited accessibility for proper oral hygiene [11]. Studies have shown that deep mucosal tunnels delay and reduce the resolution of inflammation even after adequate oral hygiene practices [12]. The depth of the mucosal tunnel represents a critical area where oral biofilms extend along prosthetic components; however, over-contoured crowns with emergence angles exceeding 30° placed on bone-level implants have also been shown to produce four times more radiographic bone loss than tissue-level implants, primarily due to patients’ reduced ability to perform adequate mechanical plaque control [13].

In esthetic areas, the vertical relationship between the IP and the CEJ of the adjacent tooth becomes particularly relevant because it influences the configuration of the interproximal restorative space and the development/maintenance of the interproximal hard and soft tissue complex [6,14].

Typically, dental implants are positioned 3 to 4 mm apical to the CEJ [15]. The determination of the ideal vertical implant position relative to the CEJ of adjacent teeth remains a cornerstone of prosthetic-driven implantology, balancing esthetic requirements with biological limitations. Historically, consensus guidelines, such as the “comfort zone” concept articulated by Grunder et al., have advocated for positioning the implant platform approximately 2 to 3 mm apical to the CEJ of adjacent teeth; however, variations in the clinical scenario in terms of hard and soft tissue levels may necessitate different apicocoronal placements and there is not yet consensus or scientific evidence investigating the topic [16,17].

While the CEJ represents a stable radiographic and esthetic landmark that allows a reproducible vertical estimation of implant position relative to the adjacent tooth, it does not necessarily reflect the actual hard and soft tissue levels of the neighboring sites, which are the most significant clinical reference points for implant placement. In this context, the vertical distance between the implant platform and the interproximal bone level of the adjacent tooth may provide additional biological information and represent an additional radiographic landmark. Furthermore, the CEJ of the adjacent teeth is also used as a landmark to diagnose the altered passive eruption, a common condition which may compromise the clinical and aesthetic outcome of the rehabilitation.

From the available scientific literature, it remains unclear whether vertical variations in implant positioning lead to increased marginal bone loss or other biological complications following functional loading, and to date, a systematic synthesis of the evidence on this topic has not yet been performed.

The aim of this systematic review was to assess the influence of baseline vertical implant positioning relative to adjacent teeth on peri-implant clinical and radiographic outcomes. Specifically, whether the radiographic vertical distance between the implant platform and the cemento-enamel junction of the adjacent tooth (IP–CEJ), and when available, the vertical distance between the implant platform and the interproximal bone level of the adjacent tooth (IP-ABL), were associated with clinical and radiographic peri-implant parameters after functional loading.

The null hypothesis was that baseline radiographic vertical implant position relative to adjacent teeth, expressed as vertical IP–CEJ distance and vertical IP–ABL distance, is not associated with peri-implant marginal bone loss or other peri-implant parameters after at least 12 months.

## 2. Materials and Methods

This systematic review adhered to the Preferred Reporting Items for a Systematic Review and Meta-Analysis (PRISMA) guidelines (Supplementary Materials-File S1). The protocol was designed before starting the study and was registered on the International Prospective Register of Systematic Reviews (PROSPERO) database (CRD420251181583). Ethical approval was not required for this study because it is a systematic review and meta-analysis based exclusively on previously published data. The Grading of Recommendations, Assessment, Development, and Evaluations approach (GRADE) was used to rate the quality of the evidence.

The focused question of this systematic review was as follows: “Does the baseline vertical implant platform position relative to adjacent teeth affect clinical and radiographic peri-implant parameters?”

Inclusion criteria were formulated according to the PECOS criteria (population, exposure, comparison, outcome, study type) to select the most eligible studies.

Population (P): Partially edentulous patients rehabilitated with implant-supported prostheses.

Exposure (E): Baseline vertical implant positioning relative to adjacent teeth (expressed as IP–CEJ distance and IP-ABL distance and measured radiographically).

Comparison (C): Different baseline values of vertical implant positioning relative to adjacent teeth analyzed as a continuous variable.

Outcomes (O): The primary outcome was the measurement of peri-implant marginal bone loss between baseline and at least 12 months of follow-up. Secondary outcomes extracted from the included studies, when available, comprised implant survival and success rate, prevalence of mucositis and peri-implantitis.

Study type (S): Clinical human studies, including randomized controlled trials (RCTs), prospective and retrospective studies with a minimum of 10 participants. To specifically address the focused question, a selection of studies was carried out according to the following inclusion criteria: clinical human studies including at least 10 participants; radiographic marginal bone loss recorded after at least 12 months of follow-up; studies reporting baseline vertical implant positioning relative to adjacent teeth; detailed information on the clinical protocol. In the case of multiple publications on the same patient cohort, only the one with the longest follow-up and/or the most comprehensive data was included. Studies not fulfilling the above-listed criteria were excluded. Marginal bone loss was measured radiographically as the linear distance (in millimeters) from the implant platform to the first bone-to-implant contact, comparing baseline and follow-up radiographs.

### 2.1. Search Strategy

A systematic search was conducted independently and in duplicate by two authors (P.D.A., G.D.R.) using the following electronic databases: PubMed, Cochrane Central Register of Controlled Trials, Scopus and Web of Science. The search strategy aimed at identifying peer-reviewed publications in English up to 18 January 2026 (Appendix A). The manual search was done in duplicate by two authors (P.D.A., G.D.R.) screening the following journals: *Journal of Dental Research*, *Journal of Clinical Periodontology*, *Journal of Periodontology*, *International Journal of Periodontics & Restorative Dentistry*, *Clinical Oral Implants Research*, *Clinical Implant Dentistry and Related Research*, *International Journal of Implant Dentistry*, *International Journal of Oral Implantology*, and *The International Journal of Oral & Maxillofacial Implants*.

### 2.2. Study Selection and Data Extraction

After removing duplicates, title and abstract screening was conducted by two review authors (P.D.A., G.D.R.). Subsequently, a thorough full-text analysis was carried out in all the studies deemed suitable for inclusion. The collected data were extracted using a specifically designed MS Excel spreadsheet (version 2024) (G.G., A.S.). All the steps reported above were performed independently and in duplicate by the two authors, and, in case of disagreement among them, a third senior author (R.P.) was consulted. The inter-reviewer reliability (Cohen’s kappa) of the full-text analysis was calculated. The authors filled the data extraction form highlighting the key features of each included study: study reference, year, study design, sample size, intervention and comparison, surgery details, outcome parameters, and length of the observation period.

### 2.3. Quality Assessment

The quality of each included study was evaluated by two independent reviewers in duplicate (P.D.A., A.S.), and conclusions were drawn through discussion. If disagreements occurred during the quality assessment, a third investigator (L.R.) reviewed the study, and a conclusion was reached through discussion. Single-arm clinical studies were assessed as described by the Institute of Health Economics (IHE) checklist by Moga et al., 2012 [18], classified according to the percentage of applicable criteria answered “Yes”. (Table 1).

### 2.4. Statistical Analysis

The implant was considered the statistical unit of analysis. All statistical analyses were conducted using random-effects meta-analysis models, based on the heterogeneity between the studies. In the presence of a limited number of studies, pooled estimates and their confidence intervals were calculated using the Hartung–Knapp–Sidik–Jonkman (HKSJ) adjustment, which provides more conservative and reliable inference in meta-analyses with small numbers of studies. Between-study heterogeneity was assessed using I2. Publication bias could not be assessed. Formal tests of funnel plot asymmetry (e.g., Egger’s test) are considered unreliable when fewer than ten studies are included, as recommended by the Cochrane Handbook, and the same limitation applies to the visual inspection of funnel plots. Funnel plots are nevertheless reported for each pooled outcome for transparency and completeness, and no interpretation is derived from them. Sensitivity analyses were conducted using a leave-one-out approach. An exploratory meta-regression was performed and, consistently with the same criterion, is reported descriptively without inferential interpretation. The significance level for all statistical analyses was set at 0.05. All analyses were conducted using RStudio (version 4.3.1).

### 2.5. Certainty of Evidence

The certainty of the obtained evidence was evaluated following GRADE [19].

## 3. Results

### 3.1. Literature Search Results

**Figure 1:**
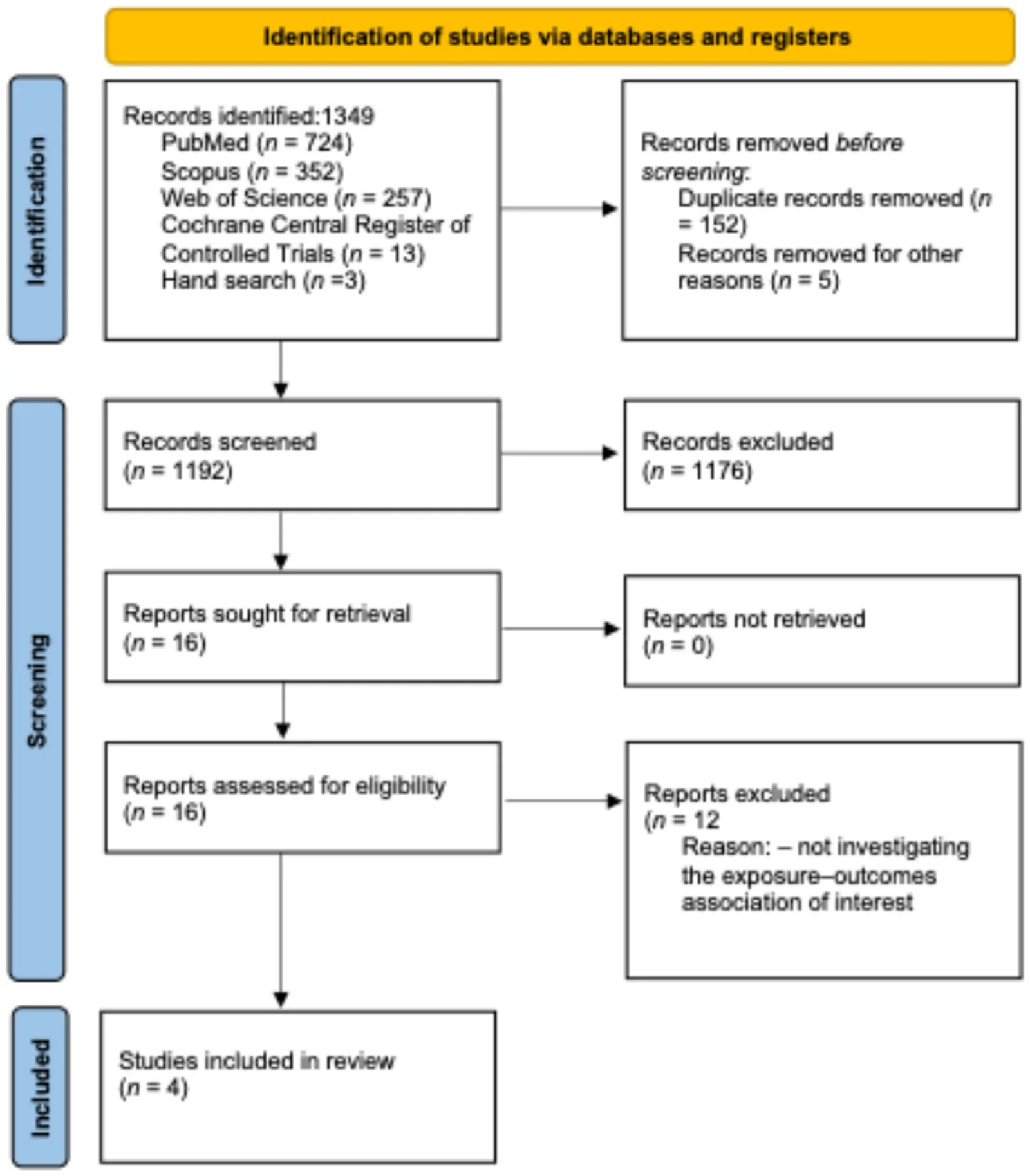
PRISMA flow diagram illustrating the study selection process.

Electronic searches initially identified a total of 1349 articles. Hand search identified three additional studies. After the removal of duplicate articles, 1192 articles were screened. 1176 of them were excluded after careful analysis of titles and/or abstracts. Full texts of 16 potentially eligible articles were further assessed, and 12 of them were excluded. Finally, four studies were included in this review [17,20,21,22]. The search process and the reasons for the exclusion are shown in the PRISMA flow chart (Figure 1). The inter-reviewer reliability (Cohen’s Kappa) of the full-text analysis was 0.85, indicating an almost perfect agreement between the two examiners.

### 3.2. Characteristics and Outcomes of the Included Studies

The main characteristics of the included studies are reported in Table 2 and Table 3. Four retrospective clinical studies were selected [17,20,21,22]. The follow-up period across the included studies ranged from 12 months to 96 months. Among all the studies, two authors reported adjunctive regenerative procedures: Mailoa et al. reported simultaneous bone regeneration, while Lin et al. reported previous bone regeneration (i.e., ridge preservation and vertical ridge augmentation) [17,20]. Three studies measured the vertical IP-CEJ and IP-ABL distances while Lin et al. measured only the vertical IP-ABL [17,21,22]. Different timepoints were considered as baseline across the included studies, and it should be noted that the 1-year interval was not anchored to the same event across these three studies. Mailoa et al. considered the implant placement as baseline, and Lin et al. the implant uncovering [17,20]. Two authors used prosthesis delivery as the reference timepoint [21,22]. To improve the comparability of outcomes, follow-up duration was standardized as much as possible for the analyses by considering 1-year follow-up data for three studies [20,21,22], while one study reported a mean follow-up of 4.42 years [17]. All the studies reported different anatomical implant locations. Chang et al. included only implants placed in the upper anterior area; Mailoa et al. and Lin et al. reported implant placement in posterior sites [17,20,21]. In contrast, one author did not specify implant position [22]. A total of 267 implants were assessed. Implant placement timing in relation to tooth extraction was reported in two articles; both authors excluded immediate implant placement and evaluated implants placed in healed sites [17,20]. In contrast, the other authors did not specify the timing of implant placement in relation to tooth extraction. All the studies reported prosthetic loading after osseointegration. Lin et al. reported the use of implants with diameters ranging from 3.25 to 5 mm and a length ranging from 8.5 to 11 mm [20], while Chang et al. reported the use of implants with a platform diameter of 4.5 mm [21]; in this study, the authors did not mention the length. The other studies reported the use of standard implants without specifying information about the exact length and diameter [17,22]. Across the included studies, different implant connections and types of restoration were used. Cardaropoli et al. [22] described the use of Brånemark standard implants with an external hexagonal connection, whereas Chang et al. and Lin et al. used implant systems with an internal conical connection [20,21]. Mailoa et al. used several types of implants and connections in the study sample [17]. Cardaropoli et al. reported screw retained restoration while Chang et al. reported cemented restorations; the other studies did not specify whether the restorations were cemented or screwed [21,22]. Cardaropoli et al. analyzed implant-supported fixed prostheses, while the other studies analyzed implant-supported single restorations [22]. A maintenance or follow-up program was reported in two studies with regular hygienic maintenance [17,20]. The remaining studies did not describe any maintenance or recall protocol. Other outcomes such as biological and mechanical complications were not reported in any of the included studies.

### 3.3. Risk of Bias

The quality appraisal was performed with the Moga et al. checklist [18] and is shown in Table 4. None of the studies was excluded because of a concerning quality appraisal; three of them were deemed to be of moderate quality [20,21,22], while one of them was of high quality [17].

### 3.4. IP-CEJ at Baseline

**Figure 2:**
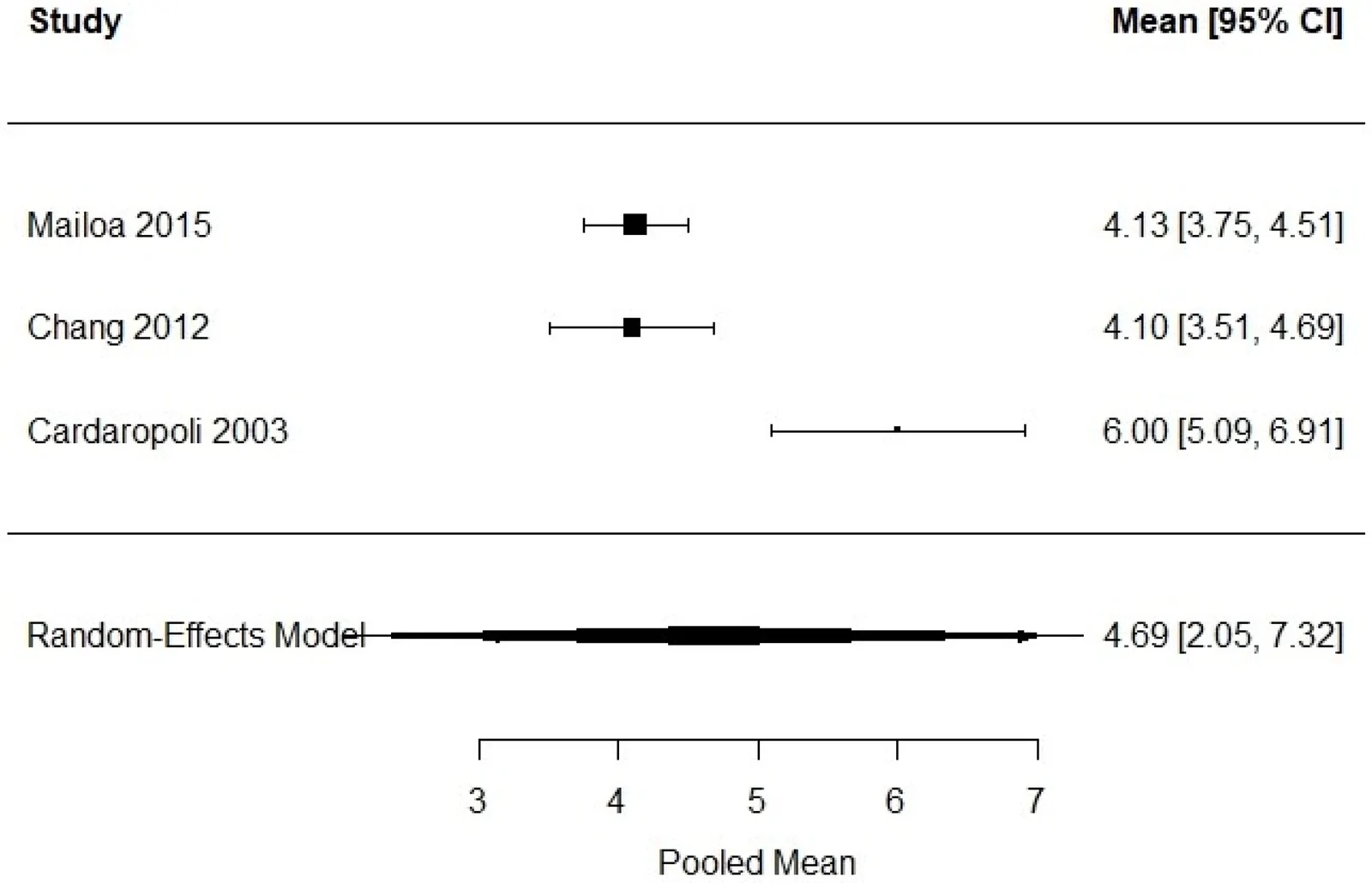
Random-effects forest plot using HKSJ adjustment showing pooled IP-CEJ vertical distance at baseline [17,21,22].

**Figure 3:**
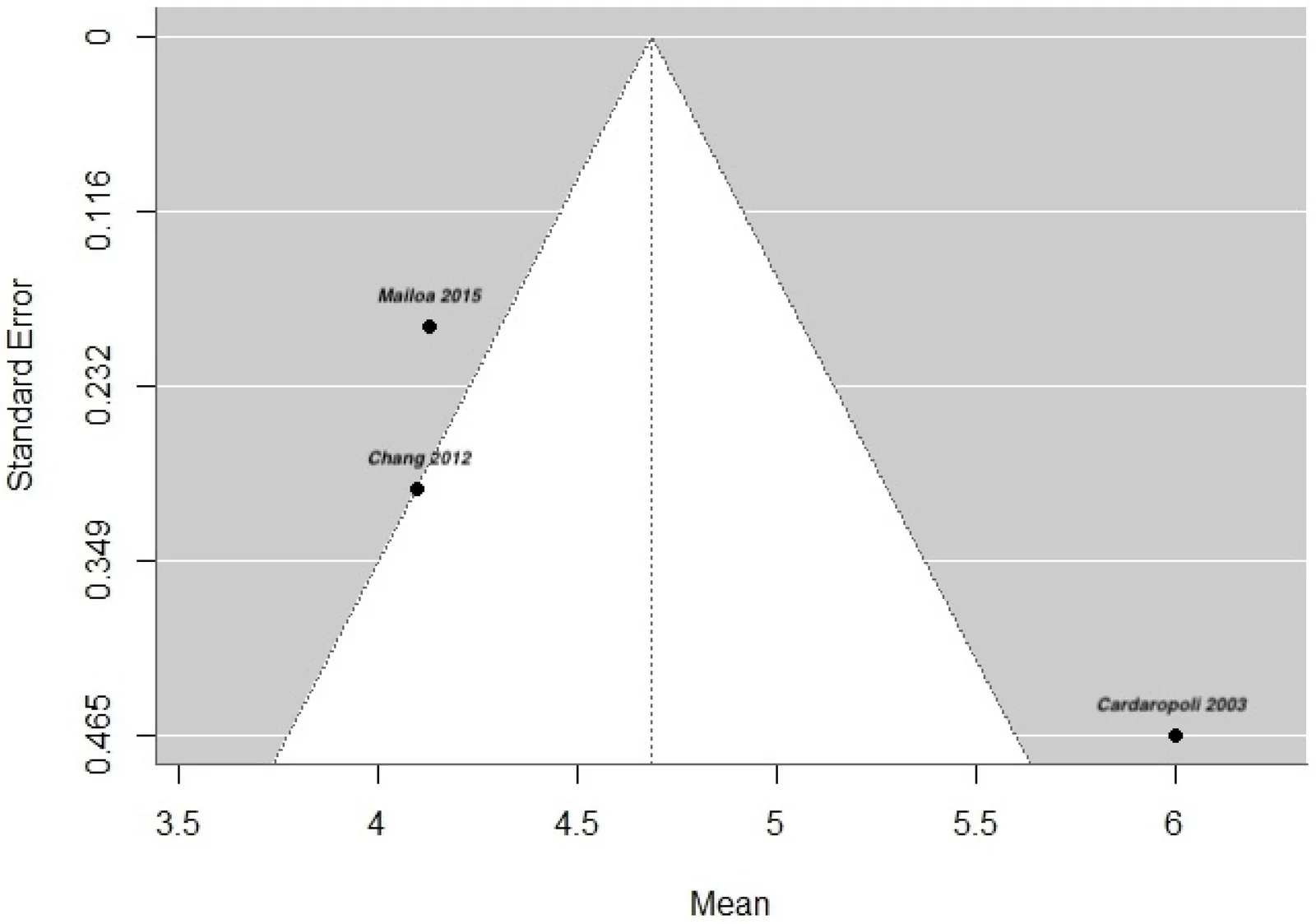
Funnel plot for baseline IP-CEJ. The dotted vertical line represents the pooled mean estimate. With fewer than ten studies, the funnel plot cannot be meaningfully interpreted; the figure is provided for transparency only [17,21,22].

Three studies contributed to this analysis [17,21,22]. The pooled mean was 4.69 mm (95% CI 2.05 to 7.32) with considerable heterogeneity (I^2^ = 91.47%) (Figure 2 and Figure 3). The leave-one-out analysis showed that the pooled mean was stable, ranging from 4.12 mm to 5.02 mm, while heterogeneity varied substantially across subsets. Notably, excluding Cardaropoli et al. reduced heterogeneity to 0%, indicating that the between-study inconsistency is largely driven by differences between Cardaropoli et al. and the other two studies.

### 3.5. IP-ABL at Baseline

**Figure 4:**
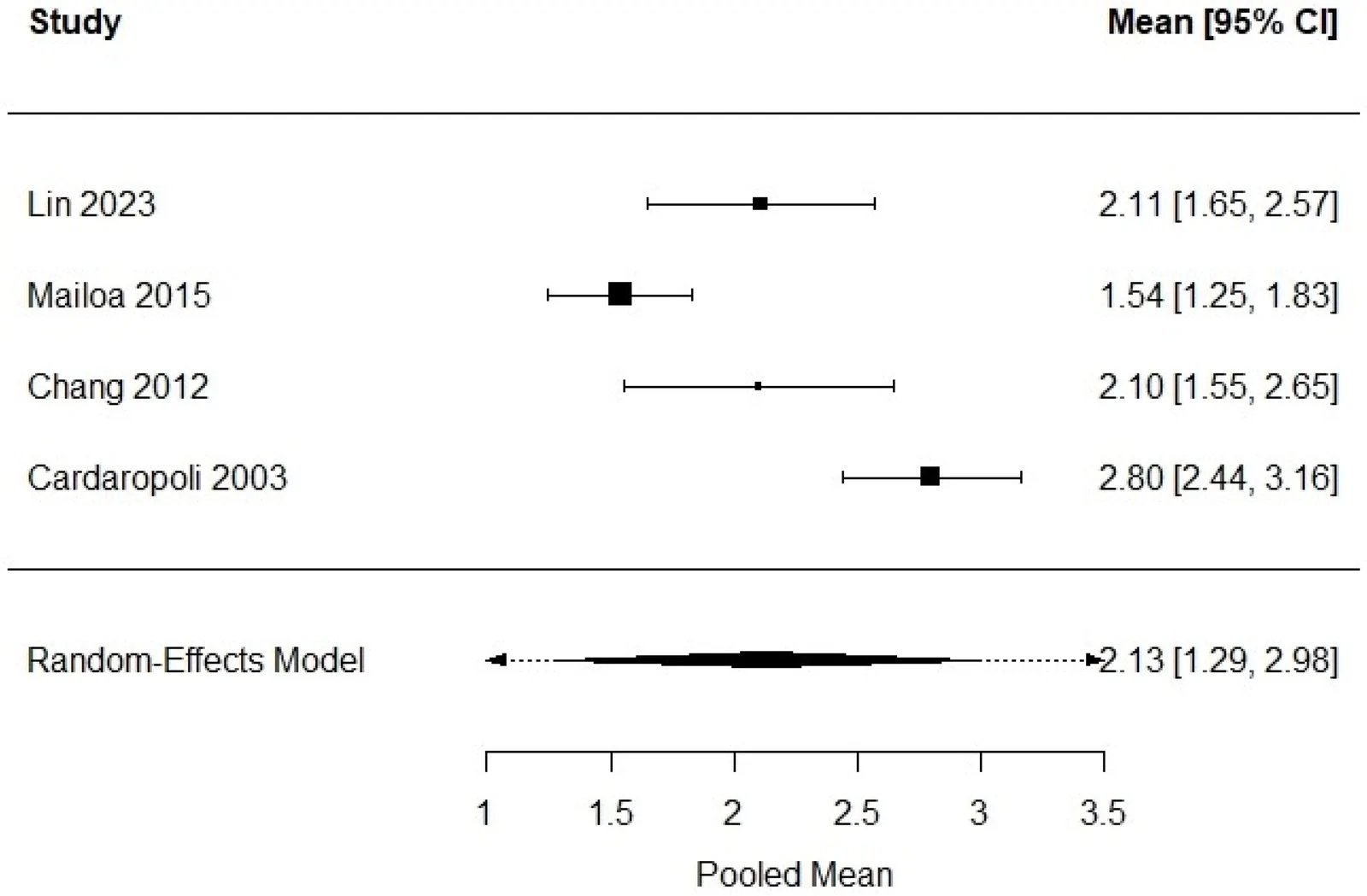
Random-effects forest plot using HKSJ adjustment showing pooled IP-ABL vertical distance at baseline [17,20,21,22].

**Figure 5:**
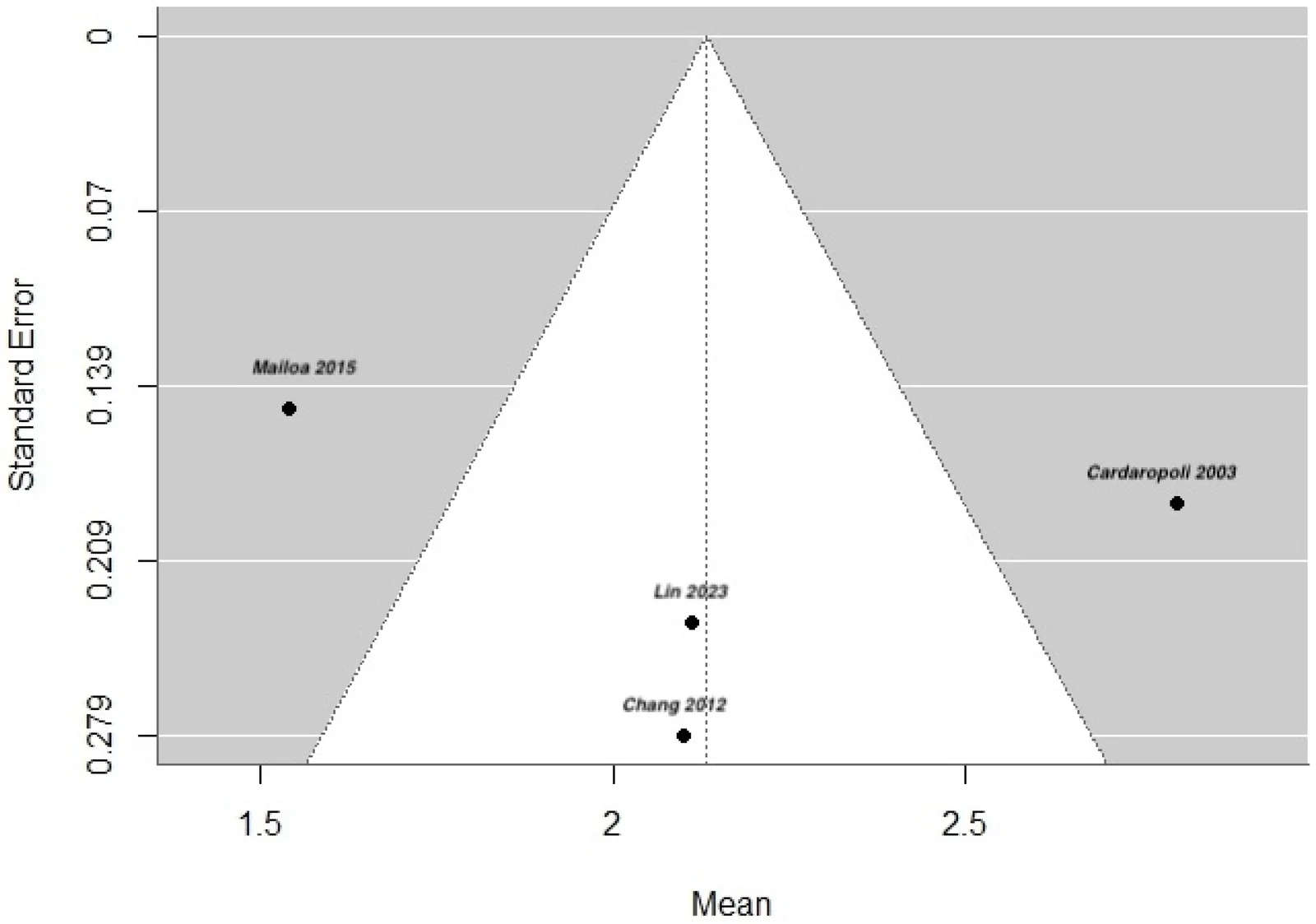
Funnel plot for baseline IP-ABL. With fewer than ten studies, the funnel plot cannot be meaningfully interpreted; the figure is provided for transparency only [17,20,21,22].

Four studies contributed to this analysis [17,20,21,22]. The pooled mean was 2.13 mm (95% CI 1.29 to 2.98) with considerable heterogeneity (I^2^ = 86.10%) (Figure 4 and Figure 5). The leave-one-out analysis showed that the pooled mean was stable, ranging from 1.87 mm to 2.36 mm. Heterogeneity varied across subsets but remained moderate to high (I^2^ = 63.95–91.59%).

### 3.6. MBL

**Figure 6:**
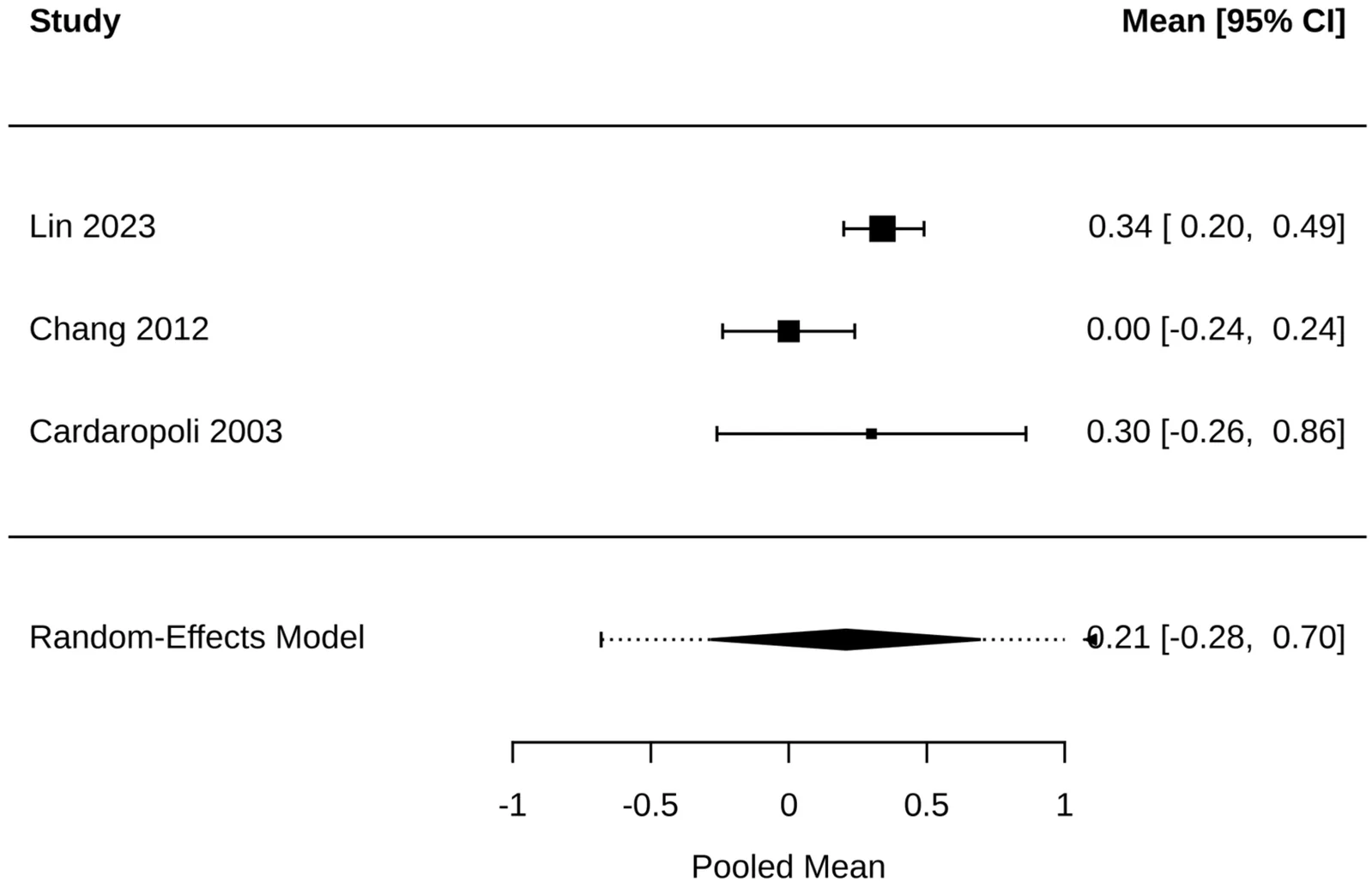
Random-effects forest plot using HKSJ adjustment showing pooled MBL measured at a timepoint at least 12 months after implant placement [20,21,22].

**Figure 7:**
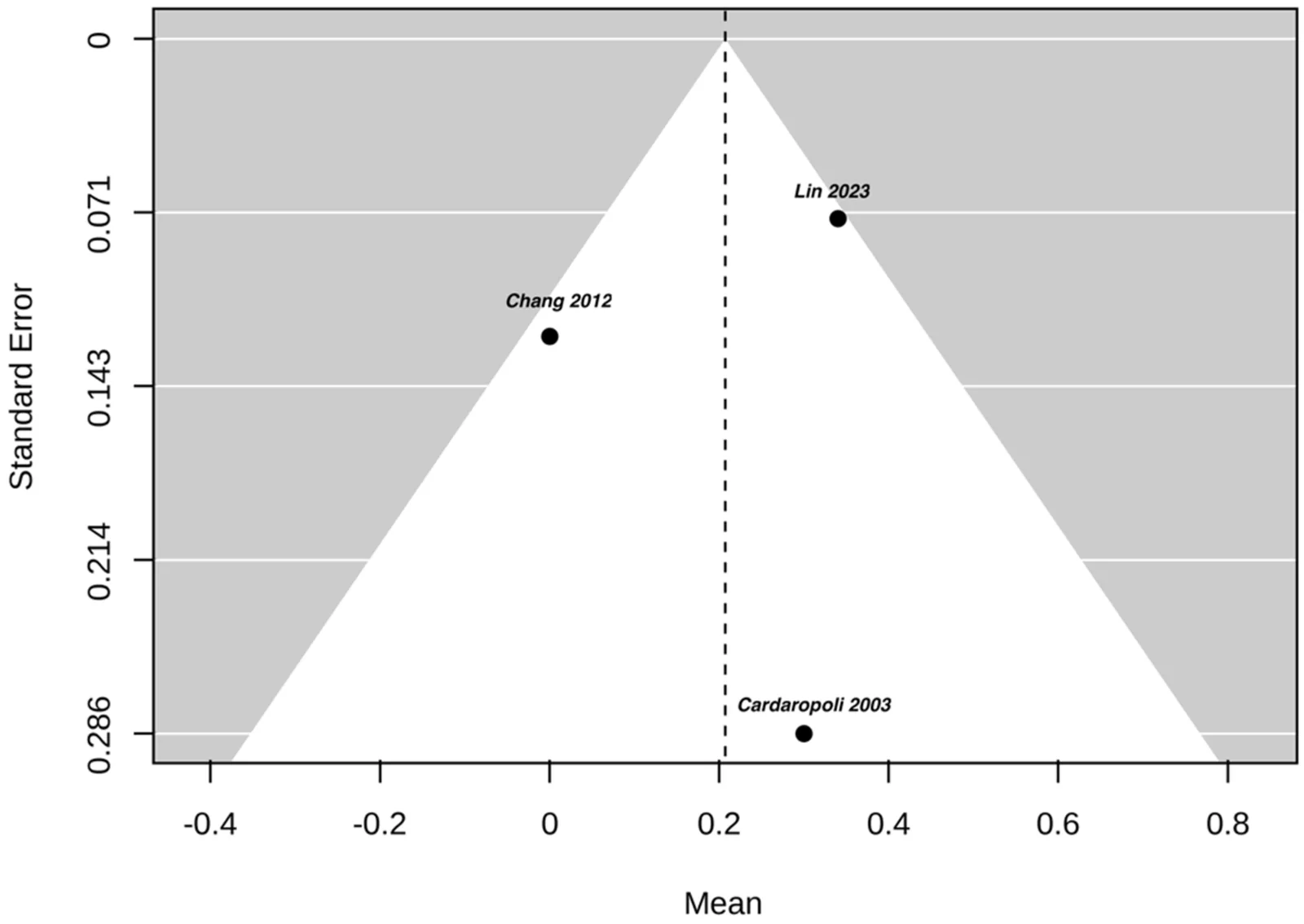
Funnel plot for MBL. With fewer than ten studies, the funnel plot cannot be meaningfully interpreted; the figure is provided for transparency only [20,21,22].

Three studies contributed to this outcome [20,21,22]. Mailoa et al. [17] was not included in this analysis because the study reported peri-implant bone level and because its observation period (mean 4.42 ± 2.5 years) did not align with the 1-year observation interval adopted for the remaining studies. The pooled mean was 0.21 mm (95% CI −0.28 to 0.70) with substantial heterogeneity (I^2^ = 63.85%; τ^2^ = 0.030; Q = 5.79, *p* = 0.055) (Figure 6 and Figure 7). The prediction interval ranged from −0.68 mm to 1.09 mm. The leave-one-out sensitivity analysis showed that the pooled estimate was stable, ranging from 0.05 mm to 0.34 mm.

### 3.7. Meta-Regression

**Figure 8:**
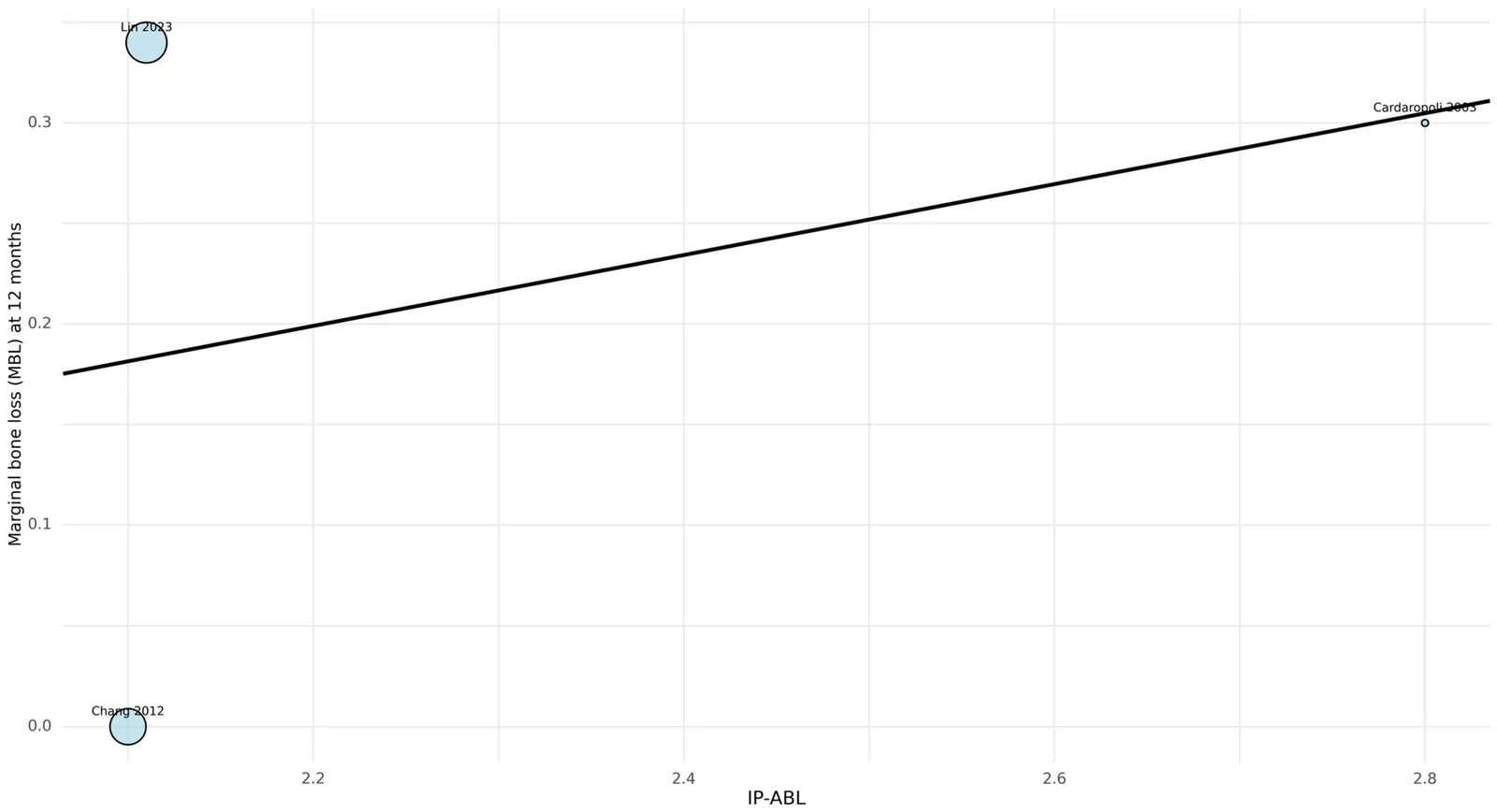
Bubble plot showing the association between baseline IP-ABL vertical distance and MBL after at least 12 months [20,21,22].

An exploratory meta-regression of baseline IP–ABL distance on MBL after at least 12 months could be fitted on three studies [20,21,22]. The estimated slope was 0.18 mm per mm of IP–ABL, and the moderator did not account for any of the between-study variability (R^2^ = 0%) (Figure 8). Residual heterogeneity remained substantial (τ^2^ = 0.047, I^2^ = 73.2%). The model is severely underpowered, and the resulting confidence interval is not clinically interpretable. Consistent with the criterion adopted for tests of funnel plot asymmetry, no inferential interpretation of this model is proposed: the analysis is descriptive only and can neither support nor exclude an association between baseline vertical implant positioning and short-term marginal bone loss.

### 3.8. Certainty of Evidence

The certainty of evidence for marginal bone loss was rated as very low according to GRADE (Table 5).

## 4. Discussion

The aim of this systematic review was to assess the influence of baseline radiographic vertical implant position relative to adjacent teeth and peri-implant clinical and radiographic parameters. Pooled data from the meta-analysis showed a mean baseline IP–CEJ distance of 4.69 mm while the mean baseline IP–ABL distance was 2.13 mm, with heterogeneity across the included studies. The association between baseline radiographic vertical implant position relative to adjacent teeth and peri-implant marginal bone loss could not be adequately tested. No meta-regression on baseline IP–CEJ distance was estimable, and the exploratory meta-regression on baseline IP–ABL distance, based on three studies, was severely underpowered. The null hypothesis could therefore not be rejected, although this reflects the limitations of the available evidence rather than evidence of no association.

Several factors have a direct impact on the IP-CEJ and IP-ABL distances, such as the hard and soft tissue levels of the adjacent tooth and of the implant site, the type of implant system used (bone-level vs. tissue-level), the implant–abutment connection design and dimension. The proper 3D implant positioning and the features of the abutment and the prosthetic restoration will allow the development of the implant supracrestal complex, maintaining a stable and healthy biological architecture of the peri-implant region [23,24]. In addition to the IP–CEJ distance, the IP-ABL distance may represent a biologically relevant parameter. While the CEJ serves as a stable interproximal radiographic and esthetic reference for prosthetic planning, the interproximal bone level directly describes the local tissue architecture and the biological support of the adjacent tooth, serving as a biological radiographic reference. Therefore, evaluating both measurements allows a comprehensive radiographic assessment of vertical implant positioning relative to the interproximal area and the surrounding dentition, which should be integrated with the soft tissue levels and clinical attachment level analyses [15]. During the planning, all the evaluations of the interproximal sides should be integrated with the buccal analysis investigating the vertical distance between the future peri-implant mucosal margin (at the most apical point) and the implant platform. The mucosal margin is a more dynamic parameter that may change rapidly over time as a consequence of several causes such as soft tissue phenotype, surgical trauma, soft tissue maturation and recession, inflammation, and prosthetic contouring. In contrast, the CEJ and the ABL constitute relatively stable radiographic landmarks and may also be associated with the height of the transmucosal tract, with the space available for the emergence profile, and provide adjunctive indications about the need for prosthetic or regenerative compensation on the interproximal side. However, it should be acknowledged that the radiographic assessment of both the CEJ and ABL, when based on two-dimensional imaging, may be affected by factors such as angulation, projection, and image distortion, leading to potential measurement inaccuracies. Alveolar bone volume reduction is an unavoidable consequence of tooth loss and pathologic processes, with variable degree and severity [25]. In case of a clinically relevant vertical alveolar bone reduction and absence of clinical attachment loss on the adjacent teeth, the soft tissues may stabilize approximately at the same level as the gingival margin of the adjacent tooth, thereby increasing the height of the transmucosal tract [12]. An increased mucosal tunnel depth, when not properly addressed through appropriate surgical and prosthetic management and patient-specific risk control measures, may contribute to the development of a dysbiotic peri-implant environment and limit the resolution of inflammation [26]. Another consideration is that the depth of the peri-implant mucosal tunnel will be even more increased on the interproximal side compared to the bucco-oral sides, especially in case of soft tissues with a pronounced scalloping. Chan et al. demonstrated that deep mucosal tunnels significantly delay and reduce inflammation resolution even after adequate oral hygiene procedures [12]. Another clinical scenario may be characterized by a significant vertical alveolar bone reduction associated with soft tissues at the implant site located apically compared to those of the adjacent tooth, which may also present clinical attachment loss. If the adjacent teeth are not affected by clinical attachment loss and maintain stable bone levels, the vertical IP-CEJ and IP-ABL distances may be reduced through staged or simultaneous vertical bone regeneration. The regenerative approach may be considered the gold standard in case of success, but vertical regeneration is an operator-dependent procedure, has a significant morbidity for the patients, requires longer treatment time and higher costs, and is associated with a high risk of complications [27]. In the presence of clinical attachment loss or when vertical bone regeneration is not performed or declined by the patient, an increased vertical IP–CEJ distance may be prosthetically compensated during the restorative phase. In such cases, this compensation is typically associated with a normal IP–ABL distance and peri-implant mucosal tunnel. However, in the worst-case scenario, characterized by vertical alveolar bone loss and clinical attachment loss, the increased IP–CEJ distance may also be associated with an increased IP–ABL distance and a deeper peri-implant mucosal tunnel, potentially leading to greater biological susceptibility. Prosthetic compensation has clinical and biological consequences due to the management of the increased vertical dimension, the change in the ideal prosthetic design and emergence profile, and the use of specific abutments [28]. The prosthetic compensation requires extensive horizontal and vertical surfaces to compensate for the volumetric deficiency and positions the implant–abutment interface at an apical and less favorable level in areas where there may also be a critical bone volume as well as non-optimal soft tissues [29,30]. This simplified, and bidimensional representation may be further aggravated if associated with a horizontal translation of the implant platform on the lingual/palatal side and with buccal–oral cortical bone level mismatch [31,32].

On the other hand, reduced vertical IP-CEJ and IP-ABL distances with a reduced transmucosal tract may also represent a challenging condition for rehabilitation, leading to the fabrication of a prosthetic design non-harmonious with an over-contoured emergence profile [33]. Strauss et al., in a preclinical study, showed that the emergence angle significantly affects MBL and that a wide emergence angle favors plaque accumulation and bleeding on probing [32]. In another study, Strauss et al. observed that the inflammatory response increases steadily as the width of the emergence angle increases and appears more pronounced at buccal sites than at lingual sites [34]. The same research group suggests evaluating the emergence angle in a three-dimensional manner, and not only on the distal and mesial aspects, in order to guarantee an adequate biological space, model the restoration to obtain esthetics and maintainability, and a three-dimensionally correct implant placement implies both an appropriate apico-coronal and bucco-oral position [35,36]. Another consideration is related to the effect of the vertical implant positioning on the bone level of adjacent teeth. Yeh et al. showed that an implant positioned more than 2 mm apically from the crestal bone level of the adjacent teeth is associated with greater bone loss for the teeth [37].

The long-term success of implant-supported rehabilitation therefore depends on the combination of several factors that ensure biological stability achieved through proper management of the available spaces, such as the transmucosal space [38]. In 2025, Fabbri et al. [39] proposed the concept of the Biological Room as the area immediately coronal to the bone–implant interface and suggested a minimum vertical dimension of 2 mm to ensure long-term peri-implant stability. Coronal to this region lies the Restorative Room, which is essential for prosthetic integration, esthetics, and emergence profile design; the authors recommend an extension up to a maximum of 3 mm in order to ensure soft tissue stability and reduce the risk of complications [39]. In addition to respecting the Biological Room, the prosthetic protocol and the number of abutment disconnections seem to be also clinically significant. According to Molina et al., the One Abutment One Time protocol reduces interproximal bone loss around implants, while also enhancing papilla preservation and the stability of the surrounding soft tissues [40]. In this regard, the choice of prosthetic components also plays an essential role, as demonstrated in a randomized clinical trial by Galindo-Moreno et al., which clinically showed that implants restored with abutments ≥ 2 mm in height exhibited significantly less marginal bone loss than those restored with shorter abutments [41,42]. Our analysis could not establish whether IP-CEJ and IP-ABL distances contribute to the magnitude of early bone changes. This aligns with the general understanding that the early phase of marginal bone remodeling is dominated by the initial healing of the peri-implant tissues and the establishment of the soft tissue supracrestal complex [39]. During this phase, several other factors, including surgical protocol, implant design, surface and connection, hard and soft tissue phenotypes, hygiene control and accessibility, functional load, multiple disconnections, and prosthetic features, seem to play a much stronger role than the initial vertical relationship with the adjacent teeth [41,43,44]. The absence of a detectable association is most likely explained by the very small number of studies available for the quantitative synthesis, which leaves the analysis without the power to identify even a moderate effect. The short follow-up period may represent an additional explanation, since the vertical relationship with the adjacent teeth could plausibly become relevant only over longer observation periods.

In modern dentistry, the scientific literature consistently affirms that long-term implant success depends on a complex interaction between biological factors and technical variables. The identification of the biological risk profile and control of local risk factors are critical aspects for achieving a successful rehabilitation. Therefore, contemporary implant rehabilitation should be based on careful risk assessment, planning, and surgical-prosthetic protocols aimed at preserving and boosting the biological potential of hard and soft tissues, in order to optimize prognosis, ensure long-term success, and harmoniously integrate within the oral environment [44,45].

Despite the relevance of the findings, several limitations must be acknowledged when interpreting the results. First, the number of studies included is small, which limits the robustness of the results. In addition, the observational retrospective nature of the studies as well as the substantial clinical heterogeneity reduces the comparability across studies and jeopardizes the possibility of drawing causal inferences. Specifically, the current literature lacks studies that evaluate in an integrated manner the combined effect of vertical and horizontal implant platform positioning, implant angulation on the buccal/oral and mesial/distal plane, emergence profile and prosthetic design and components, as well as buccal bone thickness, width and height of keratinized tissue, and long-term oral hygiene maintenance. The heterogeneity of these factors complicates the isolated interpretation of results and highlights the need for future controlled prospective studies capable of analyzing these variables in a standardized and comprehensive manner. Furthermore, MBL at 12 months, although frequently investigated, represents only a surrogate outcome and cannot be considered a reliable indicator of long-term implant success (considered as the absence of technical and biological complications) and survival. These factors highlight the need for cautious interpretation of the results and underscore the importance of future research on this critical topic. Interventional and standardized multicentric studies are needed to clarify the potential role of these parameters on peri-implant tissue stability. Future investigations should also account for the confounding variables mentioned and should incorporate a comprehensive assessment.

## 5. Conclusions

Within the limitations of this systematic review, it was not possible to determine whether baseline vertical IP–CEJ and IP–ABL distances relative to adjacent teeth are associated with peri-implant marginal bone loss after at least 12 months. The available evidence is further insufficient to assess the impact of these variables on implant success and survival rates, prosthetic and esthetic outcomes and patient-reported outcome measures (PROMs), or to identify an optimal range of baseline vertical IP–CEJ and IP–ABL distances.

## Figures and Tables

**Table 1:** Quality appraisal criteria based on the Moga et al. checklist.

Total Score (% of Applicable Criteria Answered “Yes”)	Quality Rating
85–100% (17–20/20)	High quality
65–84% (13–16/20)	Moderate quality
<65% (<12/20)	Low quality

**Table 2:** Characteristics of the included studies.

Author	Study Design	N. of Patients	N. of Implants	Type of Implants	Reference Points	Follow-Up (Years)	Regenerative Procedures
Lin	Retrospective clinical study	42	60	Bone level	ABL	1 year (starting from implant placement)	Yes
Mailoa	Retrospective clinical study	139	139	Bone level/tissue level	ABL and CEJ	At least 3 years, mean 4.42 ± 2.5 years (starting from implant loading)	Yes
Chang	Retrospective clinical study	31	33	Bone level	ABL and CEJ	1–5–8 years (starting from implant loading)	NR
Cardaropoli	Retrospective clinical study	28	35	Bone level	ABL and CEJ	1–3 years (starting from implant loading)	NR

NR: Not reported.

**Table 3:** Timing of the rehabilitation workflow.

Author	Implant Placement Timing	Implant Loading	Implant Restoration
Lin	After 4–6 months	4–6 months (2 months osseointegration, 2–4 months tissue management)	NR
Mailoa	After healing	NR	NR
Chang	NR	After 6 months from the implant position	Cemented restoration
Cardaropoli	NR	Brånemark protocol (without specifying the months)	Screw-retained restoration

NR: Not reported.

**Table 4:** Quality appraisal assessed using the Moga et al. checklist.

Study	Quality Rating	Outcome
Lin	16/20—80%	Moderate quality
Mailoa	17/20—85%	High quality
Chang	15/20—75%	Moderate quality
Cardaropoli	15/20—75%	Moderate quality

**Table 5:** GRADE assessment of the certainty and strength of evidence.

Outcome	N. of Studies	Study Design	Pooled Mean (mm)	95% CI	Certainty of Evidence	Notes
Peri-implant marginal bone loss	3	observational (retrospective) studies	0.21	−0.28–0.70	Very low	risk of bias, inconsistency, and imprecision

## Data Availability

Data supporting the findings of this systematic review are available upon reasonable request to the corresponding author.

## Supplementary Materials

The following supporting information can be downloaded at: https://www.mdpi.com/article/10.3390/dj14080523/s1. File S1: The PRISMA 2020 checklist [46].

## Appendix A. Search Strategy

#1

“Dental Implants”[Mesh] OR “Dental Implants, Single-Tooth”[Mesh] OR “Dental Implant-Abutment Design”[Mesh] OR “Dental Implantation”[Mesh] OR “Dental Prosthesis, Implant-Supported”[Mesh] OR “Dental Implantation, Endosseous”[Mesh] OR “Oral Implant*”[tiab]

#2

“Tooth”[Mesh] OR “Tooth Cervix”[Mesh] OR “Adjacent tooth*”[tiab] OR “Approximal tooth*”[tiab] OR “Proximal tooth*”[tiab] OR “Vertical distance*”[tiab] OR “Vertical discrepancy*”[tiab] OR “Implant depth”[tiab] OR “Cementoenamel junction”[tiab] OR “CEJ”[tiab]

#3

“Mucositis”[Mesh] OR “Peri-Implantitis”[Mesh] OR “Bone Resorption”[Mesh] OR “Alveolar Bone Loss”[Mesh] OR “Marginal bone loss”[tiab] OR “Crestal bone loss”[tiab] OR “Peri-implant bone loss”[tiab] OR “Marginal bone level*”[tiab] OR “Bone level change*”[tiab]

#1 AND #2 AND #3
